# Late Frosts

**Published:** 1849-05

**Authors:** 


### LATE FROSTS.

The thermometer, for two or three mornings about the middle of April, fell, in the neighborhood of this city, as low as 24 deg., and the freeze has been destructive to most of the fruits and tender vegetation. The damage done to the crops of cotton, corn, and small grain in the south, is said to be very great.

